# Paddle-to-Podium: a four-phased applied research model, translating research into practice for female surfers

**DOI:** 10.3389/fspor.2025.1619283

**Published:** 2025-07-18

**Authors:** J. Parsonage, M. MacDonald, M. Shephard, S. Gosney, A. Denny, C. Minahan

**Affiliations:** ^1^ Griffith Sports Science, Griffith University, Gold Coast, QLD, Australia; ^2^ High-Performance Program, Surfing Australia, Casuarina, NSW, Australia; ^3^ Sport Performance Innovation and Knowledge Excellence (SPIKE) Research Unit, Queensland Academy of Sport, Nathan, QLD, Australia; ^4^ Female Performance and Health Initiative, Australian Institute of Sport, Canberra, ACT, Australia

**Keywords:** Olympics, sports science, high-performance teams, female athlete, performance

## Abstract

The announcement of Teahupo’o, Tahiti as the location for surfing events at the Paris 2024 Olympic Games prompted a focus on performance research specific to female surfers. Following a 14-year absence of competitive female surfing at Teahupo’o, athletes and coaches expressed concerns about safety and wellbeing due to the physical and technical demands of sprint-paddling into such challenging and dangerous waves. This paper presents a methodological research approach designed to address these critical performance challenges in the lead up to the Paris 2024 Olympic Games. The “Paddle-to-Podium” project aimed to enhance sprint-paddling performance in elite Australian female surfers, with the dual objectives of improving wave-riding performance and ensuring athlete safety during the event. A collaborative partnership was formed between Surfing Australia, Griffith University, the Queensland Academy of Sport, and the Australian Institute of Sport to establish a multidisciplinary team committed to supporting Australia’s Olympic success in elite female surfing. The team implemented a four-phase applied research model to bridge the gap between scientific insights and practical application within the daily training environment, ensuring timely and impactful high-performance outcomes. The four phases were: (1) *Explore* current perceptions of sprint-paddling performance among elite female surfers, (2) *Examine* key stroke characteristics that contribute to superior sprint-paddling velocity, (3) E*xecute* a tailored technique intervention to optimize sprint-paddling performance, and (4) S*ustain* research practices to provide a legacy for elite female surfing in Australia.

## Introduction

On March 3, 2020, the International Olympic Committee officially approved Teahupo’o, in Tahiti as the surfing venue for the Paris 2024 Olympic Games. Renowned for its powerful and dangerous waves, Teahupo’o has a history of severe consequences for those who lack the necessary physical and technical skills to surf the wave. So consequential is the wave, that to date five deaths have been recorded. Notably, in 2006, just 14 years before this announcement, the professional surfing federation decided to exclude Teahupo’o from female competitions due to concerns over the risk of serious injury. Consequently, for nearly two decades, elite female surfers have not included this wave in their training, as there was no competitive reason to do so.

It is widely recognised that to develop expert knowledge of a specific wave location, an athlete must possess not only the requisite physical attributes but also a specialized skill set cultivated through exposure, familiarity and repetition (1). At Teahupo’o, one of the most critical attributes for success is sprint-paddling velocity. Underscoring this was two-time World Champion Tyler Wright who stated: “*There is not enough information or stats on how I paddle, and the time difference in say* 0–3 s *and the amount of distance I cover*”, she added, “*The physicality side, it will come down to we simply can’t paddle fast enough*” (2). Athletes need to reach a sufficiently high velocity to match the speed of the large wave, optimizing their wave-riding potential (3). Failure to reach the sprint-paddling velocity that the wave demands can result in “going over the falls”, where the wave’s crest overtakes the surfer, projecting them into the trough before they can ride it. This is particularly dangerous at Teahupo’o where the trough comprises of up to eighteen inches of water above a shallow reef, significantly increasing the risk of severe spinal cord and traumatic brain injuries if the surfer impacts the reef (4). Similar apprehensions about elite female surfers’ preparedness for Teahupo’o was shared by other elite surfers, their performance coaches and acknowledged by Surfing Australia, the National Sporting Organization (NSO).

### Research in sprint-paddling and sport performance in female athletes

Research on sprint-paddling in surfing has predominantly focused on male athletes, examining variables such as anthropometry, upper-body strength, paddle technique, and their associations with sprint-paddling performance (5–10). These studies have yielded practically important findings for practitioners, such as the significant association between upper-body pull-up strength and sprint paddling velocity (5, 8). However, only two studies have included elite female surfers, both limited to sex-based comparisons of gross sprint-paddling metrics such as peak paddling velocity and split times (11, 12). For example, Parsonage et al. (11) reported significantly slower sprint-paddling times in female surfers over distances of 0–5 m (12.4%), 0–10 m (9.7%), and 0–15 m (10.9%), with the largest performance gap between sexes occurring in the initial 0–5 m. Despite the discrepancies across all split times, no studies have investigated any potential associations between strength, technique, and sprint-paddling performance in female surfers. This lack of investigation highlights a significant gap in the scientific literature, limiting the development of evidence-informed training practices tailored to elite female surfers. Understanding the specific performance determinants in this population is essential to support their progression in a sport where physical capacity and skill execution are critical to competitive success.

### Knowledge translation between science and practice

It is well documented that the role of the sport scientist is crucial in facilitating evidence-informed decision-making to enhance athletic performance (13). Sports scientists offer a unique skill set through their ability to assess, analyze and translate scientific data in meaningful outcomes for coaches and support staff, providing valuable insights that guide training and overall performance strategies. Bridging the gap between “science” and “practice” remains a central goal, with various research models proposed to address this translatory challenge that may occur in high-performance sport settings (14, 15). Of these models, the Applied Research Model for Sports Scientists (ARMSS) is the most frequently cited (26). ARMSS outlines an eight-stage process to optimize sport performance acknowledging that that applied research is rarely linear and often requires a bi-directional approach, with the overlapping of stages.

Bishop (14) emphasized the need for a multidisciplinary approach, integrating the expertise of varying sports scientist disciplines with the perspectives of coaches and athletes to define performance challenges. However, practical examples of applying these models in the high-performance sport settings remain scarce (16, 17). As outlined by ARMSS, the roles of the sport scientist as a practitioner and as a researcher are often treated as distinct and separate (16). Coutts (15) elaborates on this distinction through the concepts of the “fast practitioner” and the “slow researcher”. The fast practitioner operates at a rapid pace, delivering innovative and efficient data in real time to meet the immediate demands of performance environments. In contrast, the slow researcher prioritizes data that is accurate, evidence-based and ethical, but this approach is more time-consuming (15).

High-performance sport contexts often lack the resources to support both roles, leading to the emergence of a hybrid position: the “research-practitioner” (18). This role combines practical and research responsibilities, identifying the performance-driven questions and leveraging external expertise when necessary (18). Before the initiation of the Paddle-to-Podium project, this hybrid role was well-established at Surfing Australia, and its principles directly informed the design and implementation of the applied research model. The duties and responsibilities of the “research-practitioner” provided the structural and operational foundation for the project.

In summary, the decision to hold the 2024 Olympics at Teahupo’o exposed a critical gap in both preparation and scientific understanding of sprint paddling velocity in elite female surfers. While existing research has focused predominantly on male surfers, little is known about the mechanistic factors influencing sprint-paddling in elite females. To address this, a collaborative, Olympic focused research and innovation strategy— “Paddle-to-Podium”—was athlete-centered, coach-supported, research-informed project aimed to improve the sprint-paddling performance of elite Australian female surfers, ultimately contributing to Australia’s pursuit of Olympic medal success.

## Methods

### Four-phased model

**Figure 1:**
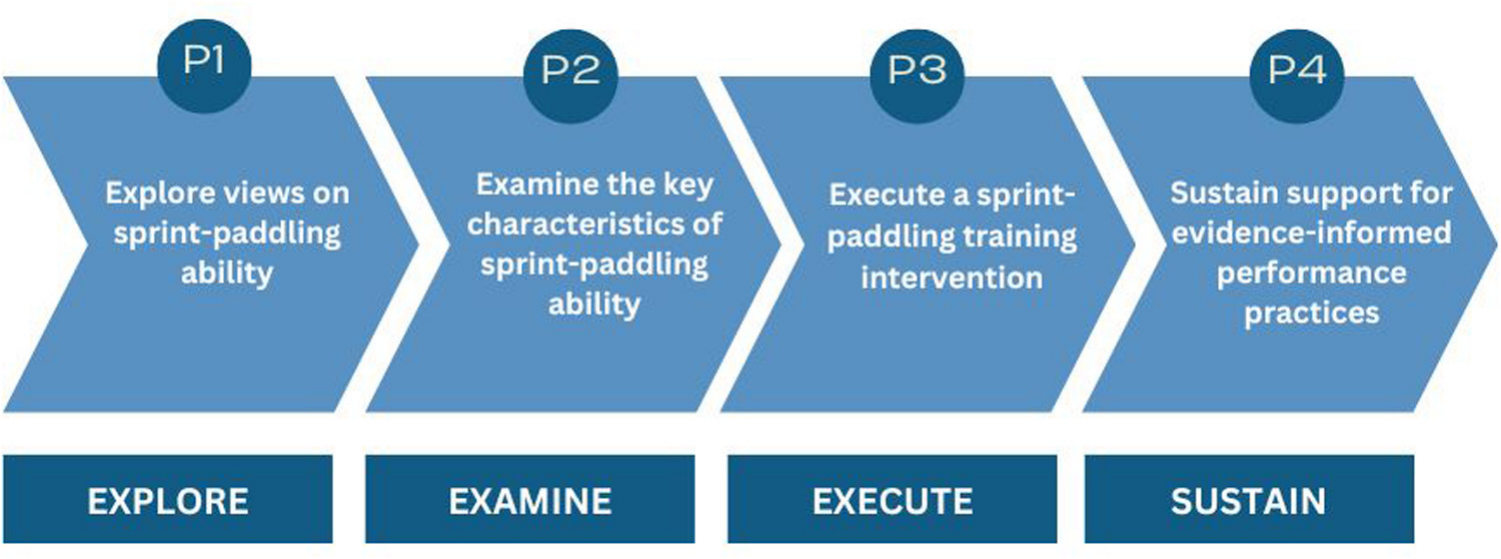
The four-phases of Paddle-to-Podium. P1-Phase One, P2-Phase Two, P3-Phase Three, P4-Phase Four.

To deliver this project, a four-phase research model was implemented, adapted from the AMRSS model (Figure 1). The model was designed to achieve the primary aim of enhancing the success of Australian female surfing athletes at the Paris 2024 Olympic Games (14). The four phases of the research model were: (1) *explore* the current perceptions of elite female surfers’ sprint-paddle ability, (2) *examine* the key characteristics that result in superior sprint-paddle velocity, (3) e*xecute* a tailored training intervention to maximise sprint-paddling performance, and (4) *sustain* support for and participation in evidence-informed performance practices to provide a legacy for elite female surfing in Australia for decades to come.

Underpinning this model, was a multi-layered theoretical framework, integrating systems theory and ecological dynamics to comprehensively address the challenge of elite female surfer preparation for Teahupo’o. While systems theory provided the overarching lens to understand the complex interactions and feedback loops between organisations, practitioners, athletes, and support staff (19, 20), ecological dynamics offered a complementary perspective focused on the athlete’s direct interaction with their performance environment (21, 22).

#### A multi-organizational collaboration

**Figure 2:**
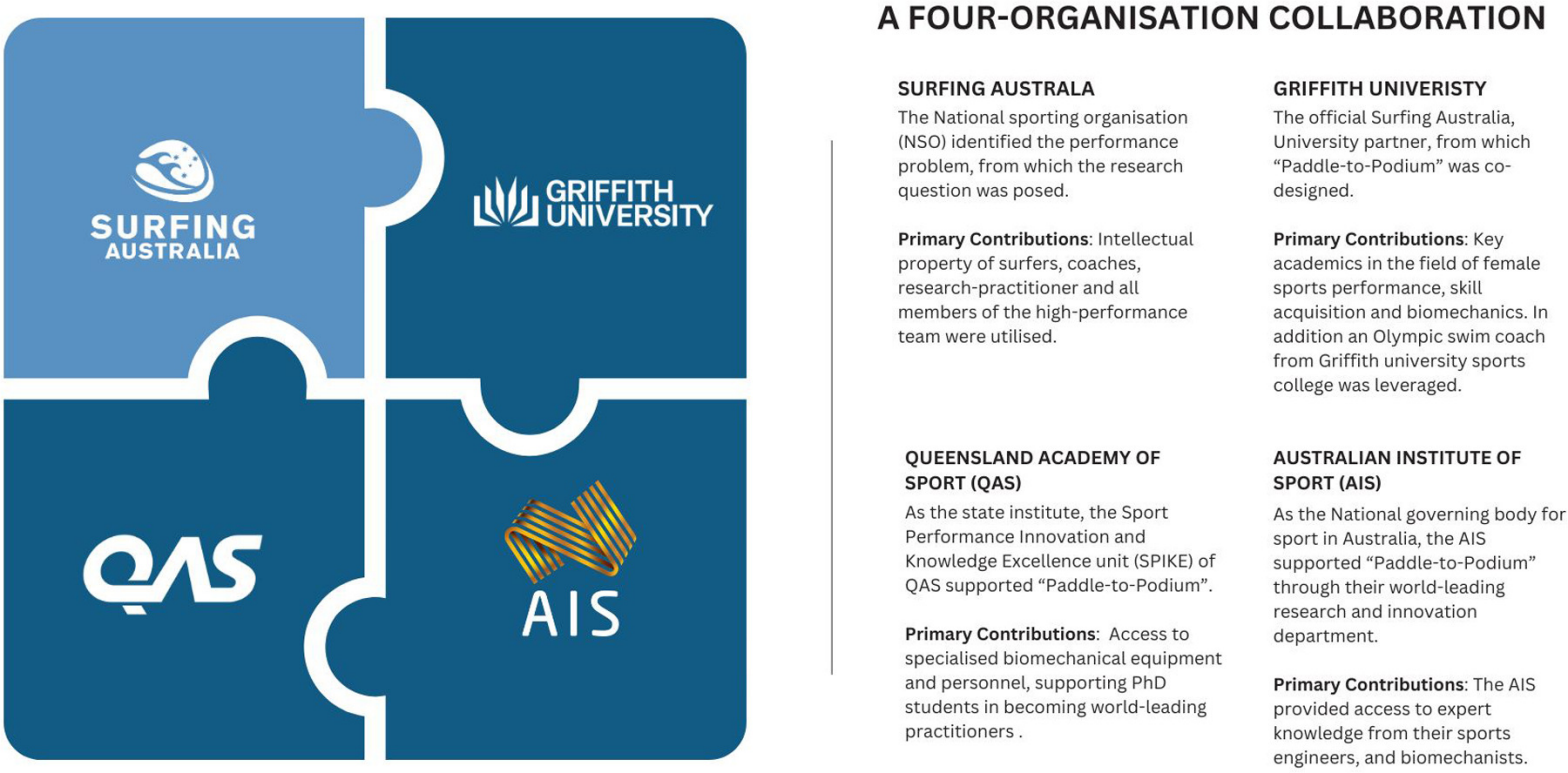
A visual representation of the four-organization collaboration approach used in the “Paddle-to-Podium” project.

A unique collaborative partnership was established between four world-leading sporting organization (Figure 2). The existing partnership between Surfing Australia and Griffith University provided the foundation for this project. Regular meetings between practitioners and researchers fostered discussions on research, practice, and innovation, facilitating early exploration of the project concept. Key university academics with expertise in female sports performance, skill acquisition, and biomechanics were engaged early in the process to ensure a robust, collaborative and scientifically sound approach across all phases.

The collaboration with the Queensland Academy of Sport (QAS) and its Sport Performance Innovation and Knowledge Excellence (SPIKE) research unit was instrumental in advancing the Paddle-to-Podium project. QAS, through SPIKE, provided access to advanced equipment and practitioner expertise, enabling refined methodological design and detailed technical analyses. Additionally, SPIKE helped to facilitate the recruitment and training of PhD students, embedding them within a high-performance environment to enhance their practical and research skills. To ensure the long-term implementation of research findings, SPIKE supported the documentation of processes and protocols, establishing a sustainable framework for ongoing innovation in female surfing performance. This strategic collaboration with SPIKE was critical in bridging research and practice for lasting impact.

Finally, as a NSO it was important to collaborate with the Australian Institute of Sport (AIS), a division of the Australian Sports Commission that provides world-class support to athletes and performance staff at a national level. It was identified that the research, innovation and technology department at the AIS, consisted of key personnel with expertise in sports engineering which was seen as advantageous to this project in relation to surfboard design and its interplay with sprint-paddle performance. Furthermore, they acted as a conjugate to innovative private industry partners that demonstrated the alignment with the project requirements.

#### Building the team

**Figure 3:**
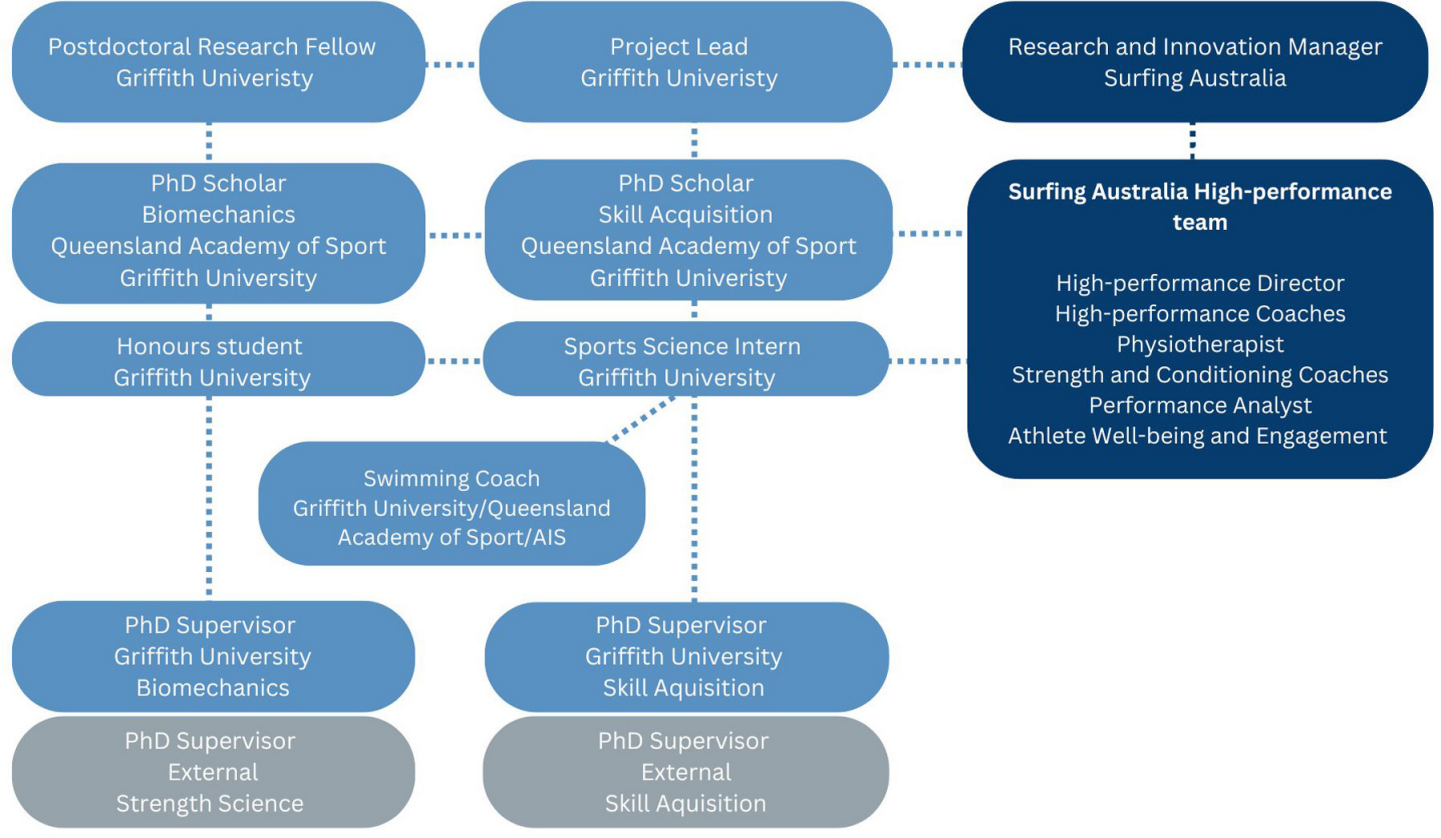
The “Paddle-to-Podium” team that was established to ensure the right capabilities and skill sets were available to the research project.

A multi-disciplinary team is defined as an integration of several specialized disciplines working together and focused on solving one common problem (23). These specialized disciplines were reflected in the “Paddle-to-Podium” team that was formed, of which the key positions across the four organizations are highlighted in Figure 3.

##### Project leadership

The leadership team consisted of a senior academic, post-doctoral research fellow and research-practitioner. The senior academic’s primary research focus was on female athlete performance, with prior publications in surfing (24, 25). Furthermore, the senior academic had extensive experience in the supervision of multiple post-doctoral fellows and PhD scholars. The post-doctoral research fellow was recruited to contribute to the intellectual thrust of the project, with a strong research background, inclusive of projects undertaken in high-performance surfing (26). Finally, the research-practitioner, who held a PhD in the sex-based differences in physical performance characteristics of elite surfers, was embedded within the research setting.

##### Scholars

Two PhD scholars were recruited and embedded within the NSO Surfing Australia. The scholars were enrolled at Griffith University, supported by the SPIKE research unit at QAS. The scholars were selected due to the expertise in biomechanics and skill acquisition, with each driving Phases 2 and 3, respectively.

##### High-performance team

Surfing Australia’s high-performance team is divided into two pillars; coaches and performance support staff, both of whom are overseen by the high-performance director. The head coach and performance support manager were the main conjugate between the surfer and research-practitioner, while aiding in supporting project timelines. Performance support staff inclusive of strength and conditioning coaches, the physiotherapist, performance analyst and athlete well-being and engagement were integral to the project’s success, utilizing their niche skill sets and facilitating athlete engagement.

##### Additional

Several key academics in applied biomechanics and skill acquisition from Griffith University and other external universities were targeted to help support the project and co-supervise PhD scholars. An Australian Olympic swimming coach from Griffith University was also engaged for Phases two and three of the projects, applying their expertise in the technical training of elite swimmers. This coach was part of the Gen32 coach initiative, supported by the QAS and AIS (27).

#### Athlete recruitment and participation

Recruitment was restricted to athletes categorized by Surfing Australia as either; highly-trained/National level (Tier 3), elite/International level athletes (Tier 4), or world-class (Tier 5) (28). Strategically, Tier 3 athletes were prioritized for piloting the intervention and conducting reliability testing of methodologies, as their larger sample size provided a robust foundation for these preliminary phases. Conversely, Tier 4 and 5 athletes—those targeted for Olympic selection—were prioritized for biomechanical analysis of paddling technique and tailored training interventions. This recruitment strategy offered several advantages (1). Availability—Tier 3 athletes, with less demanding competition schedules, were more readily available for reliability testing, (2). Minimized load—Limiting physical and psychological demands on Tier 4 and 5 athletes prevented overload during critical preparation periods, and (3). Pilot data utilization—Insights from Tier 3 athletes were used to refine protocols and improve the recruitment success of Tier 4 and 5 athletes, in later phases of the research. Finally, it is well documented that performance measures at lower levels of competition (e.g., Tier 3) typically exhibit greater within-subject variability (29). If the testing protocols demonstrated acceptable reliability with Tier 3 athletes, confidence in their applicability to Tier 4 and 5 athletes was significantly strengthened.

## Results

The following section details the four-phase methodological framework applied to the “Paddle-to-Podium” project. Each phase was designed with a clear aim, employing specific methods to generate key findings that not only addressed the immediate objectives of that phase but also informed and shaped the direction of the subsequent phases.

### Phase one: explore

Phase One aimed to explore the importance of sprint-paddling and more specifically its technique to female surfing performance. Understanding the importance of collecting valid data for the foundational phase, elite Australian surfers, surf coaches and performance support staff (i.e., strength and conditioning coaches, physiotherapists and psychologists) were interviewed using a semi-structured format (30). The primary objective was to gather insights and perspectives on sprint-paddling, while ensuring engagement with the key stakeholders from the start of the project. As such, early and strategic engagement ensured that all stakeholders were actively involved in designing and articulating the research question, resulting in a collaborative co-design approach. Through the thematic analysis of semi structured interviews, the key themes pertaining to sprint-paddle performance of elite surfers were identified and used to inform Phase Two and Phase Three. These themes included, a surfer’s paddling technique, position on the surfboard and their strength and mobility and guided the technique analysis and training interventions implemented in subsequent phases (Phase Three).

### Phase two: examination

The primary purpose of this phase was to examine the key characteristics of sprint-paddling technique that are associated with superior sprint-paddling performance. The insights gathered from semi-structured interviews with key stakeholders were analysed and used to identify the primary components of sprint-paddling technique considered essential by experts in the sport.

The initial component of this phase, aimed to examine the reliability of two different sprint-paddling assessments. Specifically, the two assessments were adapted from previously reported protocols that were reliable and valid and had been used extensively in both swimming and surfing research (31–33). This ensured that the specific protocol selected would be the most appropriate to detect meaningful performance changes in the intervention studies. A 12 s tethered test was implemented to measure sprint-paddling force production, while a 15 m sprint-paddle test was applied to measure 5 m, 10 m and 15 m split times. Both tests demonstrated good to excellent intra-session and inter-session reliability for average force and sprint-paddling split times, respectively (ICC = 0.76–0.97). Importantly, this phase had the greatest reliance on the state support partner (QAS), with the experience and knowledge of the QAS leveraged to optimize the assessment of sprint-paddling technique during the 15 m sprint paddle assessment, whilst ensuring best practice protocols were adhered to.

The key components of the assessment battery focused on evaluating the contribution of physical capacities (shoulder function and maximal shoulder strength) (34), equipment design, kinanthropometry (35), and technique (spatiotemporal characteristics) to sprint paddling performance in female surfing athletes. This broad evaluation of potential factors eliminated bias in subsequent interventions and introduced a novel focus on the interaction between surfboard design and performance, engaging typically overlooked stakeholders such as board shapers. However, the spatiotemporal analysis yielded the most meaningful and modifiable outcomes, identifying key variables that could be targeted within a technique focused intervention to enhance sprint-paddling performance in phase 3. In summary this phase achieved its intended aims, identifying the key characteristics related to sprint paddle performance through the application of valid and reliable assessments.

### Phase three: execution

Phase Three of this project focused on formulating and implementing the targeted technique training interventions aimed at enhancing the sprint-paddling performance of elite female surfers. Although a randomized control trial is considered the gold standard for research methodology, implementing them in elite athletic populations is inherently difficult due to athlete availability and sample sizes, the inability to effectively blind the athletes and researchers, and to ethically ensure that all athletes are exposed to the hypothesized “best” intervention. As outlined, a solution to balance efficacious research (does the intervention work?) with effective research (does the intervention work in practice?) is imperative (36). As a result, this phase adopted two different research methodologies, to ensure both rigorous and applicable research outcomes in a real-world setting.

The first intervention employed was a six-week, group-based training study involving Tier 3 female surfers as participants. This intervention focused on sprint-paddling technique adopting a constrains led approach to modify spatiotemporal stroke characteristics. It also incorporated a 6-week control period that preceded the training, followed by a 6-week retention period to assess whether changes in performance were sustained. The second methodology involved a single subject case study in a recently retired (within 12-months) Tier 5, elite female surfer. This case study involved a two-week intensive training period, which aligned with the typically limited timeframe available for targeted exercise-based interventions between competitions. The duration was also representative of the duration between when the Olympic event would occur following a World Championship Tour event. Importantly, these two methodological approaches aligned with the coaching and training prescription environment for these athletes. Specifically, Tier 3 athletes typically undertake individual performance planning, yet will be coached in a group setting, with some degree of individualization. Conversely, Tier 5 surfing athletes are coached on an individual basis and are prescribed highly individualized training programs.

The successful implementation and improvement of sprint-paddle performance outcomes across both research methodologies led to the adoption of the evidence-informed paddle technique training program by female Olympic surfers in preparation for the Paris 2024 Olympics. More specifically, one female Olympian outlined her want to focus on this during her pre-Olympic off-season completing a structured sprint-paddle training program under the guidance of Olympic swim coach and PhD Scholar. The rationale for the athlete’s willingness to adopt the program was three-fold. First, she recognised the critical role of sprint-paddling in her potential Olympic success and had consistently expressed this priority since the project’s inception. She had been invested in the process from the outset, though initially unsure how the research team could specifically enhance her sprint-paddling performance. Second, the athlete was able to be confident in both the assessment protocols and the performance data provided to her during Phase 2. Finally, following Phase 3, the athlete observed improvements in a peer of comparable elite status and received positive qualitative feedback from that participant, further reinforcing the program’s perceived value.

### Phase four: sustain

Phase Four aimed to strategically integrate the methodological approaches of phases two and three into daily training environment at Surfing Australia. This was achieved by establishing a national testing protocol with benchmarks for the sprint-paddle performance and the measures that were identified as significantly related to this performance, such as sprint-paddle technique. Furthermore, by ensuring constant stakeholder engagement throughout each phase, the technical sprint-paddling factors, and subsequent training interventions became regular training modalities within the daily training environment, facilitated by the surf coaches and strength and conditioning practitioners. These technique interventions are currently being implemented from the grass roots programs through to the elite athletes, highlighting “Paddle-to-Podium”s’ long-term integration and impact of applied research.

## Discussion

“Paddle-to-Podium” was a four-phased applied research project, integrating both science and practice to maximise enhance sprint-paddling performance at the Paris 2024 Olympic Games. The innovative research model aimed to enhance the opportunity for competitive success of elite Australian female surfers on the world stage. The success of which was evident, through both quantitative and qualitative performance results, strong buy-in from the world best female surfers, and the sustained impact of projects findings across the performance pathway. Furthermore, it has established a blueprint for researchers, practitioners and high-performance teams to enhance applied research translation across other high-performance sports.

The success of this project began from its inception, with the athlete identifying the “problem” and the sport posing the research question to the academics, as well as both State and National governing bodies. This led to the creation of key pillars to drive project outcomes. Firstly, it fostered strategic and targeted collaborations with organisations and leading experts, ensuring the right individuals were positioned in the right roles. It ensured consistent and authentic stakeholder engagement to maximise translational impact over a sustained period, forming long-standing and trusting relationships. It implemented an applied research framework that met high-quality research standards, while balancing the needs and realities of the high-performance daily training environment.

Phase One was instrumental in uniting all stakeholders as part of a multidisciplinary team and organically removed hierarchical structures. Based on the authors’ experience, it was frequently observed that any perception of a hierarchical structure between surf coaches, sports scientist and academics had created substantial barriers to project completion and the effective translation of research outcomes to the athletes. Moreover, research has shown how hierarchies in sport science can limit knowledge transfer by creating disciplinary silos (37). Through completing interviews with all key stakeholders, knowledge transfer was optimised and all stakeholders input valued.

Phase Two was able to analyse a broad range of factors that were associated with sprint paddle performance, through the application of reliable and valid methodologies. Although sprint-paddle technique became a focus, the other innovative and novel areas examined provide a sound foundation for further research in this area. Specifically, the interaction between athlete, their equipment and sprint-paddling performance has become a topic of conversation that is changing the landscape of surfboard shaping for females. This is something that has been observed in other sports like Tennis where equipment (i.e., string tensions, racket face size) is adjusted to an athlete’s style, strength and/or anthropometry (38).

Phase Three was unique in that it adopted a dual-method approach. In recognizing the difficulty in conducting research on truly elite populations, this approach to data collection was crucial in facilitating the progression of this project. The use of single subject case studies in elite sport has become increasingly popular (39–42), as it is pragmatic for high-performance practitioners and demonstrates high ecological validity with the typical training environment. Halperin (42) also acknowledges that case studies can help bridge the research to practice gap and can aid in strengthening relationships between scientists and coach. This was evident in “Paddle-to-podium”, with the results of the case study becoming the catalyst for Olympic athlete and coach buy-in.

Phase Four outlined qualitative sustainable outcomes that this project and research model has created. The application of translational research within a high-performance setting is imperative to ensure that knowledge is gained, and the potential performance advancements are maintained and further progressed. Continuous engagement throughout the project ensured that surf coaches and strength and conditioning staff are confidently implementing the training methods as routine practice—securing the project’s sustainability.

It would be remis not to acknowledge this in one of the few applied research projects in the literature that focuses solely on the elite female athlete. The underrepresentation of research on female surfers reflects a broader trend within sports science literature concerning female athletes. In 2012, the proportion of female-focussed research in the British Journal of Sports Medicine, American Journal of Sports Medicine, and Medicine and Science in Sport and Exercise was alarmingly low at 5, 2, and 14%, respectively (43). A decade later, in 2022, these numbers showed only marginal improvement, rising to 7, 3, and 14% (44). Moreover, research on female athletes has predominantly focused on injury risk and incidence, particularly anterior cruciate ligament injuries (45, 46), the female athlete triad (47, 48), and more recently the menstrual cycle (49, 50). In contrast, studies focusing on enhancing the athletic performance of female athletes remain scarce. Encouragingly, a growing number of researchers are working to shift this historical narrative (51–54). Sonnier et al. (55) found that studies with female first authors were over four times more likely to focus exclusively on female athletes, while senior female authors were twice as likely to do so. This was mirrored in the current female centric research team, who possessed the expertise and passion to drive female performance at the elite level.

In summary, “Paddle-to-Podium” demonstrated that when research is embedded into practice with precision, intention and collaboration, it can create an immediate competitive edge while establishing a sustainable model for success in high-performance sport. This paper presents a methodological research approach designed to address these critical performance challenges at the most elite level of female sport. The “Paddle-to-Podium” project was successful in enhancing sprint-paddling performance in elite Australian female surfers, providing a legacy for years to come.

## Data Availability

The raw data supporting the conclusions of this article will be made available by the authors, without undue reservation.

## References

[1] Dann R Duhig S Roberts L Kelly V Renshaw I Headrick J. A principled approach to skill acquisition in competitive surfing: embracing representative learning design. Int J Sports Sci Coach. (2024) 19(3):2534–47. 17479541241279044

[2] STAB. Tyler, Tati, and Caroline discuss a potential women’s event at Teahupo’o (2020). Available online at: https://stabmag.com/stabcinema/tyler-tati-and-caroline-discuss-a-potential-womens-event-at-teahupoo/ (Accessed April 25, 2025).

[3] Secomb JL Sheppard JM Dascombe BJ. Time–motion analysis of a 2-hour surfing training session. Int J Sports Physiol Perform. (2015) 10(1):17–22. 10.1123/ijspp.2014-0002 24806868

[4] Nathanson AT. Surfing Injuries. Adventure and Extreme Sports Injuries: Epidemiology, Treatment, Rehabilitation and Prevention. London: Springer (2012). p. 143–72.

[5] Coyne JO Tran TT Secomb JL Lundgren L Farley OR Newton RU Association between anthropometry, upper extremity strength, and sprint and endurance paddling performance in competitive and recreational surfers. Int J Sports Sci Coach. (2016) 11(5):728–35. 10.1177/1747954116667111

[6] Coyne JO Tran TT Secomb JL Lundgren LE Farley OR Newton RU Maximal strength training improves surfboard sprint and endurance paddling performance in competitive and recreational surfers. J Strength Cond Res. (2017) 31(1):244–53. 10.1519/JSC.0000000000001483 27253832

[7] Farley OR Secomb JL Parsonage JR Lundgren LE Abbiss CR Sheppard JM. Five weeks of sprint and high-intensity interval training improves paddling performance in adolescent surfers. J Strength Cond Res. (2016) 30(9):2446–52. 10.1519/JSC.0000000000001364 26849794

[8] Sheppard JM McNamara P Osborne M Andrews M Oliveira Borges T Walshe P Association between anthropometry and upper-body strength qualities with sprint paddling performance in competitive wave surfers. J Strength Cond Res. (2012) 26(12):3345–8. 10.1519/JSC.0b013e31824b4d78 22290522

[9] Ekmecic V Jia N Cleveland TG Saulino M Nessler JA Crocker GH Increasing surfboard volume reduces energy expenditure during paddling. Ergonomics. (2017) 60(9):1255–60. 10.1080/00140139.2016.1261188 27875943

[10] Sheppard JM Osborne M Chapman DW Andrews M McNamara P. Technique adjustments influence the performance of sprint paddling in competitive male surfers. Int J Sports Sci Coach. (2013) 8(1):43–51. 10.1260/1747-9541.8.1.43

[11] Parsonage JR Secomb JL Tran TT Farley OR Nimphius S Lundgren L Gender differences in physical performance characteristics of elite surfers. J Strength Cond Res. (2017) 31(9):2417–22. 10.1519/JSC.0000000000001428 27043303

[12] Parsonage J Secomb JL Nimphius S Farley OR Lundgren L Tran TT The meaningful use of sprint paddling data to determine surfer’s strengths and weaknesses: a gender comparison. Proceedings of the Australian Strength and Conditioning Association International Conference. Gold Coast, Australia (2015).

[13] Secomb JL. Interdisciplinary sport science in individual sports-a framework for implementation. Strength Cond J. (2024) 46(1):82–9. 10.1519/SSC.0000000000000789

[14] Bishop D. An applied research model for the sport sciences. Sports Med. (2008) 38:253–63. 10.2165/00007256-200838030-00005 18278985

[15] Coutts AJ. Working fast and working slow: the benefits of embedding research in high-performance sport. Int J Sports Physiol Perform. (2016) 11(1):1–2. 10.1123/IJSPP.2015-0781 26752203

[16] Steele J Fisher J Crawford D. Does increasing an athletes’ strength improve sports performance? A critical review with suggestions to help answer this, and other, causal questions in sport science. J Trainol. (2020) 9(1):20. 10.17338/trainology.9.1_20

[17] Kirk C Clark DR Langan-Evans C Morton JP. The physical demands of mixed martial arts: a narrative review using the ARMSS model to provide a hierarchy of evidence. J Sports Sci. (2020) 38(24):2819–41. 10.1080/02640414.2020.1802093 32783581

[18] Jones B Till K Emmonds S Hendricks S Mackreth P Darrall-Jones J Accessing off-field brains in sport; an applied research model to develop practice. Br J Sports Med. (2019) 53(13):791–3. 10.1136/bjsports-2016-097082 28818959

[19] McLean S Robertson S Salmon P. Complexity and systems thinking in sport. J Sports Sci. (2024) 43(1):1–5. 10.1080/02640414.2024.2388428 39287074

[20] Reid C Stewart E Thorne G. Multidisciplinary sport science teams in elite sport: comprehensive servicing or conflict and confusion? Sport Psychol. (2004) 18(2):204–17. 10.1123/tsp.18.2.204

[21] Brymer E Davids K. Ecological dynamics as a theoretical framework for development of sustainable behaviours towards the environment. Environ Educ Res. (2013) 19(1):45–63. 10.1080/13504622.2012.677416

[22] Immonen T Brymer E Orth D Davids K Feletti F Liukkonen J Understanding action and adventure sports participation—an ecological dynamics perspective. Sports Med-Open. (2017) 3(18):1–7. 10.1186/s40798-017-0084-1 28447331 PMC5406377

[23] Bartlett JD Drust B. A framework for effective knowledge translation and performance delivery of sport scientists in professional sport. Eur J Sport Sci. (2021) 21(11):1579–87. 10.1080/17461391.2020.1842511 33096969

[24] Loveless DJ Minahan C. Peak aerobic power and paddling efficiency in recreational and competitive junior male surfers. Eur J Sport Sci. (2010) 10(6):407–15. 10.1080/17461391003770483

[25] Loveless DJ Minahan C. Two reliable protocols for assessing maximal-paddling performance in surfboard riders. J Sports Sci. (2010) 28(7):797–803. 10.1080/02640411003770220 20473821

[26] Minahan CL Pirera DJ Sheehan B MacDonald L Bellinger PM. Anaerobic energy production during sprint paddling in junior competitive and recreational surfers. Int J Sports Physiol Perform. (2016) 11(6):810–5. 10.1123/ijspp.2015-0558 26694539

[27] Australian Institute of Sport. National Generation 2032 Coach Program (2025). Available online at: https://www.ais.gov.au/people-development/national-generation-2032-coach-program (Accessed June 16, 2025).

[28] McKay AK Stellingwerff T Smith ES Martin DT Mujika I Goosey-Tolfrey VL Defining training and performance caliber: a participant classification framework. Int J Sports Physiol Perform. (2021) 17(2):317–31. 10.1123/ijspp.2021-0451 34965513

[29] Sheppard J Osborne M Chapman D Andrews M. Anthropometric characteristics, upper-body strength, and sprint paddling performance in competitive surfers. J Strength and Cond Res. (2012) 26(12):3259–66. 10.1519/JSC.0b013e31824b4d78 22290522

[30] MacDonald LA Chalkley D Parsonage J Gosney S Webster H Minahan CL. The importance of paddling to surfing performance: insights from elite athletes, coaches, and performance support practitioners. Int J Sports Sci Coach. (2024) 19(6):2334–44. 10.1177/17479541241268674

[31] Amaro N Marinho DA Batalha N Marques MC Morouço P. Reliability of tethered swimming evaluation in age group swimmers. J Hum Kinet. (2014) 41(1):155–62. 10.2478/hukin-2014-0043 25114742 PMC4120449

[32] Cortesi M Giovanardi A Gatta G Mangia AL Bartolomei S Fantozzi S. Inertial sensors in swimming: detection of stroke phases through 3D wrist trajectory. J Sports Sci Med. (2019) 18(3):438–47. 31427865 PMC6683631

[33] Sheppard JM Nimphius S Haff GG Tran TT Spiteri T Brooks H Development of a comprehensive performance-testing protocol for competitive surfers. Int J Sports Physiol Perform. (2013) 8(5):490–5. 10.1123/ijspp.8.5.490 23319455

[34] Gosney S MacDonald L Parsonage J Denny A Keogh J Minahan C. Upper-body strength and range of motion are associated with sprint-paddling force and performance in competitive female and male surfers. J Strength Cond Res. (2025). 10.1519/JSC.0000000000005132 40440554

[35] Gosney S MacDonald L Parsonage J Worsey M Denny A Minahan C. The interplay between anthropometric profile, surfboard dimensions, and surfboard design and selection processes in competitive female and male surfers. Int J Sports Sci Coach. (2025). 10.1177/1747954125133394540510535

[36] Fullagar HH McCall A Impellizzeri FM Favero T Coutts AJ. The translation of sport science research to the field: a current opinion and overview on the perceptions of practitioners, researchers and coaches. Sports Med. (2019) 49:1817–24. 10.1007/s40279-019-01139-0 31214978

[37] Eisenmann J. Translational gap between laboratory and playing field: new era to solve old problems in sports science. Transl J Am Coll Sports Med. (2017) 2(8):37–43. 10.1249/TJX.0000000000000032

[38] Allen T Choppin S Knudson D. A review of tennis racket performance parameters. Sports Eng. (2016) 19:1–11. 10.1007/s12283-014-0167-x

[39] Bezodis NE Salo AIT Trewartha G. Lower limb joint kinetics during the first stance phase in athletics sprinting: three elite athlete case studies. J Sports Sci. (2014) 32(8):738–46. 10.1080/02640414.2013.849000 24359568

[40] Schmitt L Regnard J Auguin D Millet GP. Monitoring training and fatigue with heart rate variability: case study in a swimming Olympic champion. J Fit Res. (2016) 5(3):38–45.

[41] Barbosa AC Valadão PF Wilke CF Martins FDS Silva DCP Volkers SA The road to 21 s: a case report of a 2016 Olympic swimming sprinter. Int J Sports Sci Coach. (2019) 14(3):393–405. 10.1177/1747954119828885

[42] Halperin I. Case studies in exercise and sport sciences: a powerful tool to bridge the science–practice gap. Int J Sports Physiol Perform. (2018) 13(6):824–5. 10.1123/ijspp.2018-0185 29929417

[43] Costello JT Bieuzen F Bleakley CM. Where are all the female participants in sports and exercise medicine research? Eur J Sport Sci. (2014) 14(8):847–51. 10.1080/17461391.2014.911354 24766579

[44] Ose BM Eisenhauer J Roepe I Herda AA Vopat BG Vopat LM. Where are all the female participants in sports and exercise medicine research? A decade later. Am J Sports Med. (2025) 53(8):2022–8. 10.1177/03635465241278350 39780766

[45] Ireland ML. The female ACL: why is it more prone to injury? Ortho Clin. (2002) 33(4):637–51. 10.1016/S0030-5898(02)00028-7 12528906

[46] Renstrom P Ljungqvist A Arendt E Beynnon B Fukubayashi T Garrett W Non-contact ACL injuries in female athletes: an international Olympic committee current concepts statement. Br J Sports Med. (2008) 42(6):394–412. 10.1136/bjsm.2008.048934 18539658 PMC3920910

[47] Torstveit MK Sundgot-Borgen J. The female athlete triad: are elite athletes at increased risk? Med Sci Sports Exerc. (2005) 37(2):184–93. 10.1249/01.MSS.0000152677.60545.3A 15692312

[48] Stand P. The female athlete triad. Med Sci Sports Exerc. (2007) 39(10):1867–82. 10.1249/mss.0b013e318149f111 17909417

[49] Lebrun CM Mckenzie DC Prior JC Taunton JE. Effects of menstrual cycle phase on athletic performance. Med Sci Sports Exerc. (1995) 27(3):437–44. 10.1249/00005768-199503000-00022 7752873

[50] Brown N Knight CJ Forrest LJ. Elite female athletes’ experiences and perceptions of the menstrual cycle on training and sport performance. Scand J Med Sci Sports. (2021) 31(1):52–69. 10.1111/sms.13818 32881097

[51] Fox A Bonacci J Hoffmann S Nimphius S Saunders N. Anterior cruciate ligament injuries in Australian football: should women and girls be playing? You’re asking the wrong question. BMJ Open Sport Exerc Med. (2020) 6(1):e000778. 10.1136/bmjsem-2020-000778 32341803 PMC7173994

[52] Nimphius S McBride JM Rice PE Goodman-Capps CL Capps CR. Comparison of quadriceps and hamstring muscle activity during an isometric squat between strength-matched men and women. J Sports Sci Med. (2019) 18(1):101. 30787657 PMC6370970

[53] Elliott-Sale KJ Ackerman KE Lebrun CM Minahan C Sale C Stellingwerff T Feminae: an international multisite innovative project for female athletes. BMJ Open Sport Exerc Med. (2023) 9(4):e001675. 10.1136/bmjsem-2023-001675 37808006 PMC10551965

[54] Minahan C Newans T Quinn K Parsonage J Buxton S Bellinger P. Strong, fast, fit, lean, and safe: a positional comparison of physical and physiological qualities within the 2020 Australian women’s rugby league team. J Strength Cond Res. (2021) 35:S11–S9. 10.1519/JSC.0000000000004106 34319942

[55] Sonnier JH Coladonato C Hahn AK Paul RW Hanna AJ Windsor JT Female authorship is driving studies of female athletes: a systematic review. Am J Sports Med. (2024) 52(9):2402–6. 10.1177/03635465231203698 38288527

